# Tea saponin additive to extract eleutheroside B and E from *Eleutherococcus senticosus* by ultrasonic mediation and its application in a semi-pilot scale

**DOI:** 10.1016/j.ultsonch.2022.106039

**Published:** 2022-05-18

**Authors:** Xinyu Yang, Tingting Liu, Shuwen Qi, Huiyan Gu, Jialei Li, Lei Yang

**Affiliations:** a Key Laboratory of Forest Plant Ecology, Ministry of Education, Northeast Forestry University, Harbin 150040, China; b College of Pharmacy, Qiqihar Medical University, Qiqihar 161006, China; c School of Forestry, Northeast Forestry University, Harbin 150040, China; d Food Processing Institute, Heilongjiang Academy of Agricultural Sciences, Harbin 150086, China; e Heilongjiang Provincial Key Laboratory of Ecological Utilization of Forestry-Based Active Substances, Harbin 150040, China

**Keywords:** *Eleutherococcus senticosus*, Tea saponin, Eleutheroside B, Eleutheroside E, Ultrasonic-assisted extraction

## Abstract

The safety of ethanol in operations and its effects on human health are gradually being questioned. Under this premise, we attempted to use the natural surfactant tea saponin, which originates from the processing residues of camellia oil, as the additive of the extraction solvent and to extract eleutheroside B and eleutheroside E in the roots and rhizomes of *E. senticosus* by ultrasonic mediation. After a single-factor experiment, extraction kinetics at different powers and reaction temperatures, and Box–Behnken design optimization, the optimal conditions obtained were 0.3% tea saponin solution as the extraction solvent, 20 mL/g liquid–solid ratio, 250 W ultrasonic irradiation power (43.4 mW/g ultrasonic power density) and 40 min ultrasonic irradiation time. Under optimal conditions, satisfactory yields of eleutheroside B (1.06 ± 0.04 mg/g) and eleutheroside E (2.65 ± 0.12 mg/g) were obtained with semi pilot scale ultrasonic extraction equipment. The experiments showed that compared with the traditional thermal extraction process, the extraction time is significantly reduced at lower operating temperatures.

## Highlights

• Use ultrasonic-assisted extraction of eleutheroside B and E from *E. senticosus.*
• Tea saponin was first added to extract eleutherosides in the process.
• Dynamic models under powers, temperatures were considered.
• Determine optimum conditions of eleutheroside B and E by response surface method.
• A 200-fold expansion of the semi-pilot test was carried out.

## 1 Introduction

*Eleutherococcus senticosus* is a multibranched deciduous shrub plant belonging to Araliaceae, and it has a height of 1–6 m, and its first- and second-year branches are usually densely thorny [1]. *E. senticosus* grows naturally in forests and shrubs and can adapt to growth at altitudes of several hundred meters to 2000 m. *E. senticosus* is naturally distributed in northeastern China, the Korean Peninsula, and the southern Amur region of Russia, Primorsky Krai and southern Sakhalin Island [2], [3]. The scale of cultivation in China is increasing each year, and the cultivation area is also expanding each year with 32,067 ha cultivated in Heilongjiang Province alone, the northernmost province in China, with a reserve of nearly 280,000. *E. senticosus* is very popular in China; its young leaves can be eaten directly as vegetables, mature leaves and fruits are brewed as tea drinks, and its roots and rhizomes are used as healthy food and traditional Chinese herbal medicine, in which roots and rhizomes have been used for thousands of years in China [4]. *E. senticosus* has also been introduced and cultivated in Europe, and its roots and fruits are used as utilized parts. Modern pharmacological studies have shown that *E. senticosus* has a variety of pharmacological effects, such as improving peripheral blood circulation [5], anti-ischemia–reperfusion injury [6], anti-fatigue [7], immunomodulation [8], [9], promoting wound healing [8], preventing bone loss [10], [11], preventing and delaying neuronal apoptosis [12], [13], and enhancing and restoring spatial memory [12], [14]. Research on the chemical constituents of *E. senticosus* started early, and a wide variety of components are known. According to incomplete statistics, it contains phenylpropanoid glycoside [15], [16], coumarin [17], phenolic acid [18], [19], flavonoid [19], [20], [21], volatile oil [22], [23] and polysaccharides [24]. The main active components of *E. senticosus* are considered to be eleutheroside B and eleutheroside E [25], [26].

**Fig. 1:**
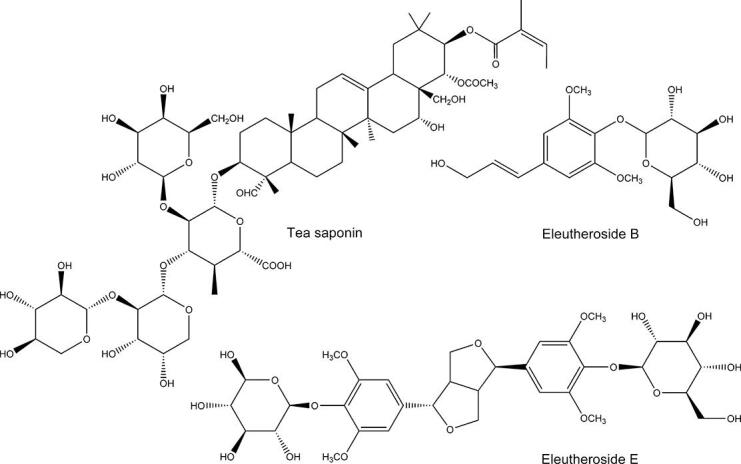
Structural formulas of eleutheroside B and E and typical tea saponins.

Eleutheroside B, also known as syringin, is a phenylpropanoid glycoside compound (Fig. 1) with a wide range of pharmacological activities, such as anti-inflammatory [27], anti-hyperglycemic [28], prevention of acute kidney injury [29], neurological [30] and cardiovascular protection activities [31]. Eleutheroside E is a lignan compound formed by the condensation of two phenylpropanoid units and is a disglycoside (Fig. 1). Eleutheroside E also has extensive pharmacological activities, including anti-lipogenesis [32], treatment of diabetic nephropathy [33], treatment of rheumatoid arthritis [34], treatment of postmenopausal osteoporosis [35], neuroprotection [36], [37] and anti-ischemia–reperfusion injury [38]. In recent years, with the continuous expansion and deepening of research on the action mechanism of eleutheroside B and eleutheroside E, extracts with eleutheroside B and eleutheroside E as index components have been favored by China, Japan, South Korea, and Southeast Asian countries.

Ultrasonic-assisted extraction is a new technology that was developed in recent years and has been successfully applied to the extraction of a variety of target analytes contained in plants [39]. It has advantages including a fast extraction speed, high efficiency, energy savings attributes and an environmentally friendly nature and is regarded as a “green technology” [39], [40]. Ultrasound is a mechanical wave with a very short wavelength, and ultrasonic-assisted extraction is a process that uses the combined effects of cavitation, vibration, grinding, stirring, etc. to destroy the cell wall and extract the cell contents [40], [41]. Ultrasonic-assisted extraction technology is especially widely used in the extraction of thermally unstable target analytes and low-temperature food processing. With the deepening of research and the continuous improvement of equipment and technology, ultrasonic-assisted extraction will be more widely used in food, medicine, the chemical industry, and other fields [39].

According to the literature, pure water [42] or moderate concentration (40%-50%) ethanol [43], [44] is usually used as the extraction solvent of eleutheroside B and eleutheroside E. When pure water was selected as the extraction solvent, the extraction yield was low due to the low solubilities of eleutheroside B and E in water. When an ethanol solution is used as the extraction solvent, since ethanol is a flammable and volatile solvent, the extraction site is subject to high fire, explosion, and environmental protection pressures, and ethanol is also likely to remain in the obtained extract. Recent studies have shown that inhalation or ingestion of ethanol can cause DNA damage and increase the risk of cancer in humans [45]. Based on this concern, it has become a general trend to seek ethanol-free extraction methods.

Tea saponins are a class of pentacyclic triterpene fractions isolated from the residue of *Camellia oleifera* oil extracts, and the chemical structure of typical tea saponins is shown in Fig. 1. The chemical structure of tea saponin contains both hydrophobic groups (triterpene skeleton) and saccharide hydrophilic units, so tea saponin is a natural nonionic surfactant. As an agrochemical, tea saponins are inexpensive and more viable for industrial use than other natural surfactants [46]. Tea saponins have been shown to be nontoxic and nonhazardous, easily assimilated by nature, nonirritating, easy to handle and use, and can replace or partially replace synthetic surfactants in some industrial uses [47], [48]. However, there is currently a lack of research on tea saponin as a natural surfactant in the extraction of poorly soluble components from plants, which seriously hinders the evaluation of the potential of tea saponins in this field of application.

The purpose of this paper is to use the natural nonionic surfactant tea saponin as an additive to extract eleutheroside B and eleutheroside E by ultrasonic treatment. The main factors affecting the yield of the target analytes, including the concentration of tea saponin, liquid–solid ratio, reaction temperature, ultrasonic irradiation power and time, were optimized by a single factor combined with the response surface method. The method was indicated to be feasible through experiments of stability, reproducibility, and recovery. In addition, to investigate the efficiency of tea saponin as an extraction additive, it was compared with conventional thermal extraction and ultrasonic-assisted extraction with water and ethanol solution as solvents. Furthermore, a semi-pilot scale attempt was performed under optimized operating conditions to evaluate the effectiveness of the technique.

## 2 Experimental

### 2.1 Materials and chemicals

The roots and rhizomes of *E. senticosus* were harvested from the Maoershan Experimental Forest Farm of Northeast Forestry University in October 2020 and were identified by Professor Gu Huiyan of this university. The roots and rhizomes were washed and air dried in a cool, ventilated place, crushed in a pulveriser, sieved to obtain a uniform powder of 40–60 mesh and stored in a sealed zipper bag at 4 ± 2 °C. Eleutheroside B and eleutheroside E were purchased from Push (Chengdu, China), and tea saponin was purchased from Yuanye (Shanghai, China).

### 2.2 High-performance liquid chromatography instruments and quantitative conditions

HPLC was used to perform the qualitative and quantitative analysis of eleutheroside B and eleutheroside E. The type of detection instrument used was an Agilent 1260 Infinity II Prime (USA). which contained a quaternary pump, a UV detector, an automatic sampler, a constant temperature column oven and an automatic degassing system. The chromatographic column was a Diamonsil C18 (250 mm × 4.6 mm × 5 μm), and the detection conditions were as follows: 0.05% trifluoroacetic acid: acetonitrile (88:12, v/v), flow rate of 1 mL/min, injection volume of 10 μL, column temperature of 30 °C, and running time of 30 min under variable wavelength detection. The detection wavelength of eleutheroside B was 265 nm, and the detection wavelength of eleutheroside E was 210 nm. Under these conditions, the peak times of eleutheroside B and eleutheroside E were 5.1 min and 22.9 min, respectively, and baseline separation was achieved between the two target peak analytes and impurity peaks. The standard curve method was used to quantify eleutheroside B and eleutheroside E, and the obtained standard curves were *Y*_eleutheroside B_ = 8258.70*x* + 5.15 (*R*^2^ = 0.9999, *n* = 7) and *Y*_eleutheroside E_ = 17304*x* − 164.84 (*R*^2^ = 0.9998, *n* = 7), and the linear ranges were 2.5–500 μg/mL and 5.0–500 μg/mL, respectively.

### 2.3 Lab scale ultrasonic extraction experiments

Lab-scale ultrasonic extraction experiments were performed in an ultrasonic bath with a maximum power of 250 W and an operating frequency of 45 kHz. The model of the bath was KQ-250DE (Kunshan, China) with internal chamber dimensions (300 × 240 × 150 mm). Liquid height of the ultrasonic bath is 80 mm, and water weight in the ultrasonic bath is 5760 g. For the extraction experiments, the ultrasonic bath was connected to a temperature-controlled bath (GDW-2015A, Changzhou, China) to control the reaction temperature. A total of 0.5 g of roots and rhizomes of *E. senticosus* powder was accurately weighed and placed in a 100 mL Erlenmeyer flask, after which a certain volume and a certain concentration of tea saponin were placed in the flask. The Erlenmeyer flask containing the starting material and extraction solvent was immersed in the ultrasonic bath so that the bottom of the flask was below the controlled water level at 10 cm from the bottom of the bath. The ultrasonic generator was turned on and allowed to be ultrasonically irradiated for a certain time at a certain ultrasonic irradiation power. After the reaction, 2 mL of the supernatant was quickly filtered out, passed through a 0.45 µm microporous membrane and then determined by HPLC, and the yields of target analytes were calculated. Parallel experiments were conducted 3 times under each operating condition, and the results were taken as the average.

To evaluate the effect of ultrasonic irradiation power and reaction temperature on the eleutheroside B and eleutheroside E yields, a first-order kinetic model was used in this study, and the relevant mathematical equations are as follows:

(1) $$\text{ln}\;\left(Y_{e}-Y_{t}\right)=\text{ln}Y_{e}-kt$$

(2) $$Y_{t}=Y_{e}[(1-\text{exp}\left(-kt\right)$$

(3) $$Y=dy/dt=kY_{e}\text{exp}\left(-kt\right)$$

where *Y_t_* and *Y_e_* represent the yield of eleutheroside B and eleutheroside E at *t* and equilibrium, respectively, *k* is the mass transfer coefficient of the process, and *Y* is the target analyte yield at any time.

Response surface methodology with three factors and a three-level Box–Behnken experimental design was fulfilled to optimize the operational parameters of the extraction process (ultrasonic irradiation power *X_1_*, ultrasonic irradiation time *X_2_* and reaction temperature *X_3_*) and to estimate the main effect, interaction effect and secondary effect of the yields of eleutheroside B and eleutheroside E. The sequence of experiments was carried out randomly to be as objective as possible and to reduce human influence. Based on the results of the one-factor experiment, the ranges of *X_1_*, *X_2_*, and *X_3_* were set to 150–250 W (ultrasonic power density 26.0–43.4 mW/g), 20–40 min and 40–60 °C, respectively, and standardized at intervals. The interaction between the response and variables was evaluated as follows:

(4).

where *Y* is the response variable; *X_i_* and *X_j_* are the independent variables, and β_0_, β_i_, β_ii_, and β_ij_ are the regression coefficients of the intercept, linearity, square, and interaction terms, respectively. By adding confirmatory experiments in subsequent experiments, the validity of the statistical experimental design adopted was verified.

### 2.4 Semi-pilot scale ultrasonic extraction experiments

Semi-pilot scale ultrasonic extraction experiments were carried out in a TGCXN-2B ultrasonic extractor (Hongxianglong, Beijing, China). The total volume of the equipment was 2.5 L, the effective working volume was 2.5 L, the ultrasonic frequency was 40 kHz, the ultrasonic irradiation power was continuously adjustable, and the maximum ultrasonic irradiation power was 1200 W (ultrasonic power density 2.08 W/g). The unit was heated by an interpolated electric heating tube with a maximum heating power of 260 W and cooling water in the outer jacket of the tank. The stirrer had a stepless speed regulation of 0–2500 r/min, and the stirring motor power was 100 W. Some of the main process parameters can be adjusted on the control panel, including ultrasonic irradiation power, ultrasonic irradiation time, stirring speed and reaction system temperature. A schematic diagram of the apparatus is shown in Chen et al. [49]. One hundred grams of roots and rhizomes of *E. senticosus* powder was used per batch (i.e., a 200-fold increase in the amount of raw material compared to the previous lab study) with a liquid–solid ratio of 20 mL/g.

### 2.5 Method validation

The stability of the target analytes was fulfilled under the optimum conditions (0.3% tea saponin concentration, 20 mL/g liquid to solid ratio, 250 W ultrasonic irradiation power, 40 min ultrasonic irradiation time and 50 °C reaction temperature). Different concentrations of target analytes were added to *E. senticosus* root and rhizome powder samples and extracted under optimal conditions to test the recoveries of target analytes. Five parallel tests (0.5 g) were performed under optimal conditions to investigate the reproducibility.

## 3 Results and discussion

### 3.1 Effect of tea saponin concentration

**Fig. 2:**
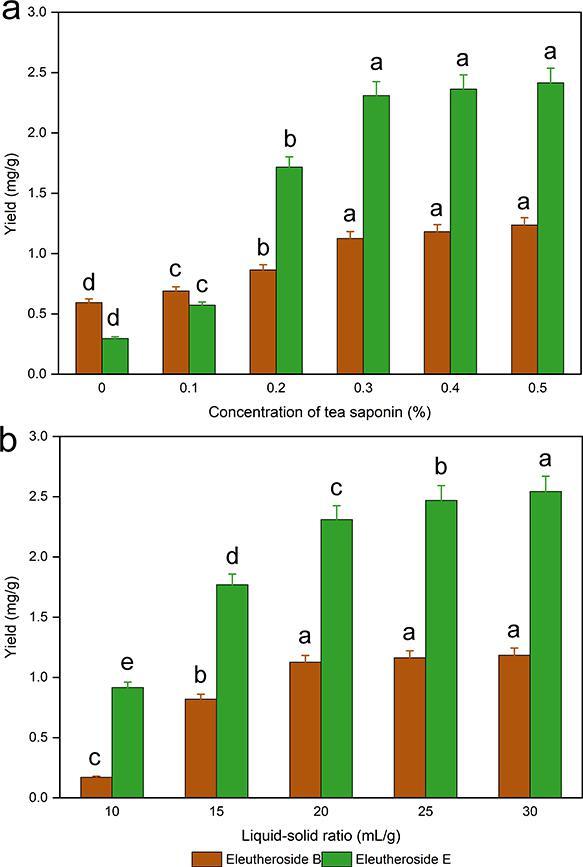
Effect of tea saponin concentration (a) and liquid–solid ratio (b) on yield of eleutheroside B and E. Values are mean ± SE (*n* = 3 replicates). Means that have different letters at the top of each bar are significantly different (*P* < 0.05).

Surfactant concentration is an important factor affecting yield. As shown in Fig. 2a, the yields of both eleutheroside B and eleutheroside E increased with increasing surfactant concentration. A significant difference (*P* < 0.05) in yields was observed between concentrations of tea saponin and liquid–solid ratios. This may be because the higher the concentration of surfactant in the solution is, the easier it is to form a micelle structure. Surfactants can form spherical micelles in water, and low-polarity substances can enter the core of the micelle or between surfactant molecules, thereby increasing the solubility of these low-polarity substances in water to achieve solubilization [50]. If the concentration of surfactant is too high, the critical micelle concentration is reached, the amount of micelle formed does not increase, and the yield does not increase. The yields of eleutheroside B and eleutheroside E showed similar trends with the change in tea saponin concentration. When the concentration of tea saponin increases from 0 to 0.3%, the yield increases rapidly, while when the tea saponin concentration further increases from 0.3% to 0.5%, the increasing trend of the yield of eleutheroside B and eleutheroside E slows down, and the inflection point appeared at 0.3% tea saponin concentration. Thus, at the lower concentration of 0.3% tea saponin, satisfactory yields can be achieved.

### 3.2 Effect of the liquid–solid ratio

The effect of the liquid–solid ratio on the yield of target analytes was investigated by experiments with different liquid–solid ratios. The liquid–solid ratio usually plays an important role in the extraction of target analytes. The results of the experiments are shown in Fig. 2b. When the liquid–solid ratio was 10 and 15 mL/g, the liquid–solid ratio was too small, and the concentration difference between the target analytes in the plant matrix and the solvent was small, which was not conducive to mass transfer. As the liquid–solid ratio increased, the difference in concentration of the target analytes between the plant matrix and the solvent increased. When the liquid–solid ratio reached 20 mL/g, the yields of both eleutheroside B and eleutheroside E tended to reach a maximum. A further increase in the liquid–solid ratio resulted in a slow increase in the yield of target analytes and a significant increase in the volume of solvent, which would increase the energy consumption of the extraction process and the difficulty of solvent recovery, resulting in unnecessary waste. Therefore, in the next optimization experiments, 20 mL/g was chosen as the appropriate liquid–solid ratio.

### 3.3 Effect of ultrasonic irradiation power and time

**Fig. 3:**
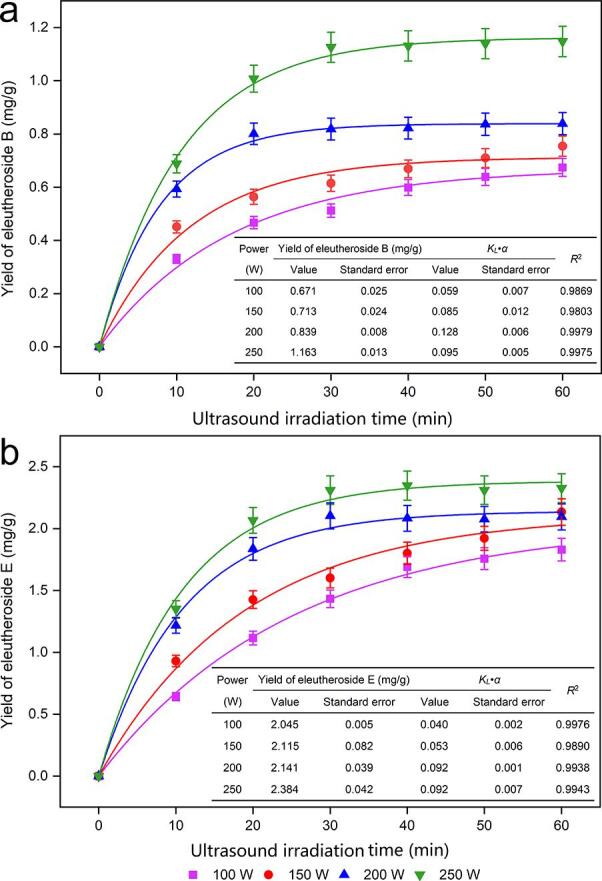
Dynamic curves for eleutheroside B and E with various ultrasonic irradiation powers (100, 150, 200, and 250 W) from roots and rhizomes of *Eleutherococcus senticosus.*

The kinetic model fits the extraction process of target analytes through mathematical equations, which can provide intuitive insight into the diffusion behavior of target analytes and is beneficial for optimization and control of the entire extraction process. Fig. 3 shows that the *R*^2^ values under different ultrasonic irradiation powers are all greater than 0.98, indicating that the first-order kinetic model can better reflect the actual experimental results. Under different ultrasonic irradiation powers, the yields of target analytes had similar trends with ultrasonic irradiation time. The yields of both eleutheroside B and eleutheroside E increased significantly within the first 30 min at the four given ultrasonic irradiation powers of the experiment, followed by little change in the yields of target analytes with further increases in ultrasonic irradiation time. Comparing the yields of eleutheroside B and eleutheroside E after reaching equilibrium, it was found that the yields of target analytes under 250 W ultrasonic irradiation power (ultrasonic power density 43.4 mW/g) were higher than the yields under other ultrasonic irradiation powers. Under an ultrasonic irradiation power of 250 W (ultrasonic power density 43.4 mW/g), the yields of the target analytes increased significantly in the first 30 min and showed a slow upward trend over 30 min. The yields of both eleutheroside B and eleutheroside E increased with increasing ultrasonic irradiation power at the same time point. It appears that the higher the power of ultrasonic irradiation was, the stronger the cavitation effect was and the stronger the breaking effect on the cell wall. However, the higher the power of ultrasonic irradiation was, the higher the energy consumption. Therefore, we chose an ultrasonic irradiation time of 20–40 and an ultrasonic irradiation power of 150–250 W (ultrasonic power density 26.0–43.4 mW/g) for further optimization.

### 3.4 Effect of reaction temperature

**Fig. 4:**
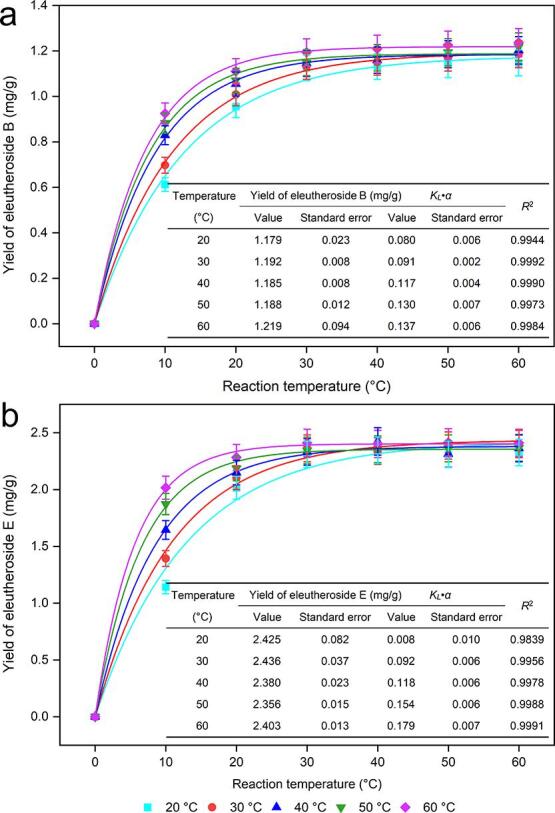
Dynamic curves for eleutheroside B and E with various reaction temperatures (20, 30, 40, 50, and 60 °C) from roots and rhizomes of *Eleutherococcus senticosus.*

As shown in Fig. 4, first, we can see that the yields of eleutheroside B and eleutheroside E extracted from the roots and rhizomes of *E. senticosus* vary with increasing reaction temperature and ultrasonic irradiation time. The yields of the target analytes increased rapidly during the first 20 min and much more slowly during the next 10 min, and when the ultrasonic irradiation time was further extended to 60 min, only a slight increase in the yields of the 2 compounds was observed. According to the correlation coefficient *R*^2^ greater than 0.98, the yields of the target analytes were well fitted by first-order kinetics at different experimental reaction temperatures, and higher yields were obtained at 60 °C. Second, the difference in equilibrium yields at different temperatures was not significant for either eleutheroside B or eleutheroside E; that is, as long as sufficient ultrasonic irradiation time is given, the yield will tend to be consistent. As seen from the temperature kinetic curve in Fig. 4, there is some interaction between temperature and time, and we chose 40–60 °C as the range for subsequent optimization.

### 3.5 Parameter optimization by response surface methodology

The seventeen experimental programs and the corresponding values of the experimental results are expressed in Table 1. Among all experiments, Groups 1 to 12 are the factorial experiments, and Groups 13 to 17 are the central experiments for the analysis of variance. The regression coefficients and variance analysis corresponding to the response surface processes of the eleutheroside B and eleutheroside E extraction experiments are expressed in Table 2. The result of variance analysis shows that the significance of the response value in the regression coefficients was determined by the F test and *P value*. The *F value*s of eleutheroside B and eleutheroside E in this model were 35.36 and 15.49, respectively, and the prob > *F* values were<0.001, and 0.0008 means that the significance of the model is relatively high. Among the factors influencing eleutheroside B, *X_1_, X_1_X_3,_ X_2_X_3_ and X_3_^2^* are extremely significant, *X_2_, X_3,_ X_1_X_2_* and *X_2_^2^* are nonsignificant, and *X_1_^2^* is highly significant; among the factors influencing eleutheroside E, *X_1_* is a highly significant term, *X_1_X_2_* is significant, *X_1_^2^, X_2_^2^, X_2_X_3_, X_1_X_3,_ X_2_* and *X_3_* are nonsignificant, and *X_3_^2^* is extremely significant. The Adj *R*^2^ values of eleutheroside B and eleutheroside E were 0.9508 and 0.8907, respectively, and the coefficients of variation (CV) were 2.22 and 2.83, respectively. It can be obtained that the model is credible. Meanwhile, the regression coefficients can not only describe the true relationship between each factor and the response value but can also be used to analyze the process of target analytes. The predicted *R^2^* values of eleutheroside B and eleutheroside E were 0.8816 and 0.7190, respectively, which indicate that the predicted value is reliable and effective. “Adeq precision” means the signal-to-noise ratio. The ratios of eleutheroside B and eleutheroside E are 20.48 and 12.71, respectively, which are greater than 4, and thus, it can be inferred that the obtained signals were adequate. The yields of eleutheroside B and eleutheroside E were given by the following equations:

*Y*_Eleutheroside B_ = − 2.2 + 1.9 × 10^-2^*X_1_* − 3.8 × 10^-2^*X_2_* + 7.4 × 10^-2^*X_3_* + 2.0 × 10^-7^*X_1_X_2_* − 1.4 × 10^-4^*X_1_X_3_* + 6.9 × 10^-4^*X_2_X_3_* − 2.4 × 10^-5^*X_1_*^2^ + 7.7 × 10^-5^*X_2_*^2^ − 6.9 × 10^-4^*X_3_*^2^.

*Y*_Eleutheroside E_ = − 6.1 + 1.3 × 10^-2^*X_1_* − 7.9 × 10^-2^*X_2_* + 3.4 × 10^-1^*X_3_* + 2.2 × 10^-4^*X_1_X_2_* − 8.6 × 10^-5^*X_1_X_3_* + 7.6 × 10^-4^*X_2_X_3_* − 3.3 × 10^-5^*X_1_*^2^ − 4.7 × 10^-5^*X_2_*^2^ − 3.5 × 10^-3^*X_3_*^2^.

**Table 1:** Experimental data and the observed response value with different combinations of ultrasound irradiation power (*X_1_*), ultrasound irradiation time (*X_2_*) and reaction temperature (*X_3_*) used in the Box–Behnken design.

No.	Experimental design			Dependent variables			
	*X_1_*: Ultrasound irradiation power (W)	*X_2_*: Ultrasound irradiation time (min)	*X_3_*: Reaction temperature (°C)	Yield of eleutheroside B (mg/g)		Yield of eleutheroside E (mg/g)	
				Predicted yield	Actual yield	Predicted yield	Actual yield
1	150	20	50	0.94	0.93	2.66	2.65
2	250	20	50	1.16	1.16	2.66	2.68
3	150	40	50	0.96	0.97	2.44	2.42
4	250	40	50	1.18	1.19	2.87	2.88
5	150	30	40	0.81	0.83	2.17	2.15
6	250	30	40	1.17	1.18	2.47	2.42
7	150	30	60	0.93	0.92	2.25	2.30
8	250	30	60	1.02	1.01	2.38	2.39
9	200	20	40	1.12	1.12	2.48	2.50
10	200	40	40	1.00	0.99	2.32	2.35
11	200	20	60	0.96	0.98	2.32	2.28
12	200	40	60	1.12	1.12	2.46	2.43
13	200	30	50	1.11	1.12	2.74	2.74
14	200	30	50	1.11	1.11	2.74	2.64
15	200	30	50	1.11	1.10	2.74	2.80
16	200	30	50	1.11	1.08	2.74	2.70
17	200	30	50	1.11	1.15	2.74	2.84

**Table 2:** Estimated regression coefficients for the quadratic polynomial model and ANOVA for the experimental results in the optimization of eleutheroside B and eleutheroside E extractions a.

Regression coefficients	Sum of squares		Degree of freedom	Mean Square		*F* value		Prob > *F*	
	*Y_B_*	*Y_E_*		*Y_B_*	*Y_E_*	*Y_B_*	*Y_E_*	*Y_B_*	*Y_E_*
Model	0.1750	0.7190	9	0.0194	0.0799	35.36	15.49	< 0.0001***	0.0008***
*X_1_*^b^	0.0991	0.0908	1	0.0991	0.0908	180.19	17.60	< 0.0001***	0.0041**
*X_2_*	0.0009	0.0001	1	0.0009	0.0001	1.69	0.01	0.2352	0.9174
*X_3_*	0.0007	0.0001	1	0.0007	0.0001	1.29	0.02	0.2928	0.9002
*X_1_X_2_*	0.0000	0.0469	1	0.0000	0.0469	0.00	9.10	0.9933	0.0195*
*X_1_X_3_*	0.0184	0.0074	1	0.0184	0.0074	33.44	1.44	0.0007***	0.2686
*X_2_X_3_*	0.0191	0.0234	1	0.0191	0.0234	34.80	4.53	0.0006***	0.0708
*X_1_^2^*	0.0149	0.0287	1	0.0149	0.0287	27.05	5.57	0.0013**	0.0504
*X_2_^2^*	0.0002	0.0001	1	0.0002	0.0001	0.45	0.02	0.5239	0.8973
*X_3_^2^*	0.0200	0.5040	1	0.0200	0.5040	36.28	97.73	0.0005***	< 0.0001***
Lack of fit	0.0011	0.0108	3	0.0004	0.0036	0.50	0.57	0.7020	0.6647
Credibility analysis of the regression equations	Index mark	Standard deviation	Mean	CV c %	Press	*R^2^*	Adjust *R^2^*	Predicted *R^2^*	Adequacy precision
	*Y_B_*	0.02	1.06	2.22	0.02	0.9785	0.9508	0.8816	20.48
	*Y_E_*	0.07	2.54	2.83	0.21	0.9522	0.8907	0.7190	12.71

* *p* < 0.05, significant; ** *p* < 0.01, highly significant; *** *p* < 0.001, extremely significant.

eleutheroside B and eleutheroside E (mg/g).

a The results were obtained with Design Expert 8.0 software.

b *X_1_* is the ultrasound irradiation power (W), *X_2_* is the ultrasound irradiation time (min), *X_3_* is the reaction temperature (°C), *Y_B_* and *Y_E_* is the yield of.

c CV is the coefficients of variation.

**Fig. 5:**
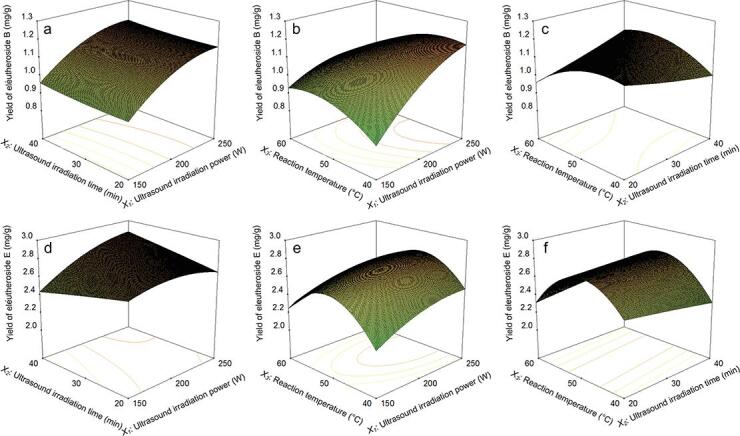
Optimization of the yield of eleutheroside B and E using Box–Behnken design. Interaction of ultrasound irradiation power and ultrasound irradiation time of eleutheroside B (a) and eleutheroside E (d), interaction of ultrasound irradiation power and reaction temperature of eleutheroside B (b) and eleutheroside E (e), interaction of ultrasound irradiation time and reaction temperature of eleutheroside B (c) and eleutheroside E (f).

The following figures show the influence of the interactions between various factors on the yields of eleutheroside B and eleutheroside E. Fig. 5a to 5f all have the maximum value of response within the investigated range. The three-dimensional response surfaces of the yields of eleutheroside B and eleutheroside E versus ultrasonic irradiation time and ultrasonic irradiation power are shown in Fig. 5(a, d). The effects of the reaction temperature and ultrasonic irradiation power on the yields of eleutheroside B and eleutheroside E are shown in Fig. 5 (b, e). The interaction of the reaction temperature and the ultrasonic irradiation time is shown in Fig. 5 (c, f). After optimizing and analyzing the experimental data through the software, the experimental conditions to reach the point prediction were as follows: the ultrasonic irradiation power was 250 W (ultrasonic power density 43.4 mW/g), the ultrasonic irradiation time was 40 min, and the reaction temperature was 50.10 °C. Under these prediction conditions, the theoretical yields of eleutheroside B and eleutheroside E were 1.18 and 2.87 mg/g, respectively.

The actual yields of eleutheroside B and eleutheroside E were 1.15 ± 0.04 and 2.85 ± 0.03 mg/g, respectively. After the results of the response surface method analysis were verified five times under the optimal prediction conditions (the ultrasonic power density was 43.4 mW/g, the ultrasonic irradiation time was 40 min, and the reaction temperature was 50 °C). The results are similar to the theoretical values simulated by the regression coefficients of the response surface method, which also illustrates that the results of response surface analysis are reliable.

### 3.6 Method validation

#### 3.6.1 Stability

Table 3 presents the results of stability tests for eleutheroside B and eleutheroside E in 0.3% tea saponin solution, from which under the optimum conditions selected, the average recovery of eleutheroside B was 98.33%, while the average recovery of eleutheroside E was 99.29%. After 7 days, the average recoveries of eleutheroside B and eleutheroside E were 95.83% and 97.86%, respectively. This shows that there was no significant degradation of either eleutheroside B or eleutheroside E under the extraction conditions obtained as previously described, indicating that the two standards were stable in 0.3% tea saponin solution.

**Table 3:** Method validation studies.

Stablity studies of eleutheroside B and eleutheroside E standards under the optimum conditions of ultrasound irradiation extraction								
Compounds	Initial concentration (mg/mL)	Recovered concentration after ultrasound irradiation extraction (mg/mL)	RSD% (n = 3)	Average recovery (%)	Recovered concentration after 7 d (mg/mL)	RSD% (n = 3)	Average recovery (%)	
Eleutheroside B	1.20	1.18	0.96	98.33	1.15	0.96	95.83	
Eleutheroside E	2.80	2.78	0.97	99.29	2.74	0.94	97.86	
Recovery of eleutheroside B and eleutheroside E from from roots and rhizomes of *Eleutherococcus senticosus* (n = 3)								
Sample	Contents of the sample (mg)		Mass of added standards (mg)		Mass of the sample analyzed with added standards (mg)		Recovery (%)	
	Eleutheroside B	Eleutheroside E	Eleutheroside B	Eleutheroside E	Eleutheroside B	Eleutheroside E	Eleutheroside B	Eleutheroside E
1	1.16	2.86	0.50	2.00	1.64	4.82	98.80	99.18
2	1.16	2.86	1.00	3.00	2.13	5.78	98.61	98.63
3	1.16	2.86	1.50	4.00	2.61	6.75	98.12	98.40
Average							98.51	98.74

#### 3.6.2 Recovery

Table 3 also lists the experimental results of the recoveries of eleutheroside B and eleutheroside E extracted from the roots and rhizomes of E. senticosus, in which the average recoveries of eleutheroside B and eleutheroside E were 98.51% and 98.74%, respectively. This experimental result is satisfactory, indicating that the ultrasonic extraction method obtained above has a high recovery of eleutheroside B and eleutheroside E in the roots and rhizomes of *E. senticosus*.

#### 3.6.3 Repeatability

The mean yields of eleutheroside B and eleutheroside E obtained from the five replicate extractions of the roots and rhizomes of *E. senticosus* samples under the optimum extraction conditions obtained as previously described showed repeatability with relative standard deviations of 3.66% and 3.93%, respectively. This result indicates that the process for the ultrasonic-assisted extraction of eleutheroside B and eleutheroside E using tea saponin solution as the solvent has excellent repeatability.

### 3.7 Comparison with conventional extraction methods

The reference conventional methods, including thermal extraction (reflux extraction with 50% ethanol [44] and hot water extraction with pure water as solvent [42]) and ultrasonic extraction (40% ethanol as solvent [43]), were compared with ultrasonic-assisted extraction using 0.3% tea saponin solution as solvent in this paper. In addition, the ultrasonic-assisted extraction method with pure water as the extraction solvent also added the contrast sequence. The yield, operating temperature, and time consumption of eleutheroside B and eleutheroside E obtained by different methods are listed in Table 4. The yields of eleutheroside B and eleutheroside E were significantly lower for both ultrasonic-assisted and thermal extraction with pure water as the solvent than for ethanol solution and 0.3% tea saponin solution as the extraction solvent; thus, it seems that the solvent plays a decisive role compared to thermal and ultrasonic cavitation. Table 4 also shows that 0.3% tea saponin solution was used as the solvent to extract eleutheroside B and eleutheroside E, and the yield was similar to that of 40% ethanol and 50% ethanol. Although the yield of 0.3% tea saponin as solvent eleutheroside B and eleutheroside E is slightly lower than that of ethanol solution, 0.3% tea saponin is a solvent mainly in the aqueous phase, which is nonvolatile, noncombustible, nontoxic, and safe. The extraction solvent ethanol solution used in conventional methods is a solvent with medium ethanol content, so the volatilization of ethanol to the environment is difficult to avoid. In contrast to ultrasonic-assisted extraction and thermal extraction, each has its own characteristics. Thermal extraction relies on increasing the speed of molecular migration and reducing solvent viscosity to accelerate the dissolution of the target analytes, whereas ultrasonic-assisted extraction relies on the cavitation effect of ultrasonic waves to increase solvent penetration, thereby increasing the diffusion of the target analytes, shortening the extraction time, and accelerating the extraction process. Compared with the two methods, thermal extraction takes a longer time and requires a higher temperature, and ultrasonic-assisted extraction takes a shorter time and requires a lower temperature. Overall, 0.3% tea saponin as an extraction solvent has obvious advantages in ultrasonic-assisted extraction of eleutheroside B and eleutheroside E from the roots and rhizomes of *E. senticosus*.

**Table 4:** Comparison of extraction by different methods.

Method	References	Solvent	Temperature (°C)	Extraction time consumption (min)	Yield (mean ± SD, mg/g)	
					Eleutheroside B	Eleutheroside E
Ultrasound irradiation extraction		0.3% tea saponin	50	40	1.16 ± 0.05	2.86 ± 0.11
Ultrasound irradiation extraction		pure water	50	40	0.64 ± 0.03	0.44 ± 0.02
Ultrasound irradiation extraction	43	40% ethanol	30	30	1.12 ± 0.07	2.79 ± 0.13
Reflux extraction	44	50% ethanol	80	120	1.22 ± 0.06	2.94 ± 0.12
Hot water extraction	42	pure water	80	210	0.87 ± 0.05	0.76 ± 0.02

### 3.8 Semi-scale verification test

The semi-scale pilot experimental study was carried out in an ultrasonic extraction tank with 2.5 L of effective volume, the ultrasonic frequency of the tank was 40 kHz, which was slightly lower than the 45 kHz of the lab-scale test. Based on the previous optimization results, the selected conditions for the scale-up pilot study were 0.3% tea saponin solution as the solvent, a liquid–solid ratio of 20 mL/g, and an ultrasonic irradiation power of 250 W at a controlled temperature of 50 °C. The particle size of root and *rhizome E. senticosus* powder used in the scale-up study (5–10 mesh) was larger than that in the lab-pilot study (40–60 mesh) to facilitate the filtration step. It has been shown that the amount and intensity of cavitation in liquids decreases at higher frequencies [51], and at constant intensity, higher amplitude values were obtained at lower frequencies. Therefore, cavitation is better at 40 kHz than at 45 kHz because of the higher amplitude, and a higher yield of eleutheroside B and eleutheroside E should be obtained as a result. However, to increase the filterability of the filter residue in the semi-pilot test process, the particle size is increased, which leads to the slow penetration of the solvent into the coarse-grained material, and the two offset each other. Compared with the lab scale, the yields of eleutheroside B and eleutheroside E were lower in semi-pilot scale extraction, which may be due to variation in particle size, frequency of operation, and shape and mixing intensity between the two systems caused by the difference. Three scale-up validation tests (100 g) were carried out on the pilot equipment. The actual yields of eleutheroside B and eleutheroside E were 1.06 ± 0.04 mg/g and 2.65 ± 0.12 mg/g, respectively.

## 4 Conclusion

In this work, we developed an efficient ultrasonic-assisted extraction method based on the natural surfactant tea saponin solution for the extraction of eleutheroside B and eleutheroside E from roots and rhizomes of *E. senticosus*. After single-factor experiments, extraction kinetics at different powers and reaction temperatures, and Box–Behnken design optimization, the optimal conditions obtained were as follows: 0.3% tea saponin solution as the extraction solvent, 20 mL/g liquid–solid ratio, 250 W ultrasonic irradiation power (ultrasonic power density 43.4 mW/g) and 40 min ultrasonic irradiation time. Satisfactory yields of eleutheroside B (1.06 ± 0.04 mg/g) and eleutheroside E (2.65 ± 0.12 mg/g) were obtained with semipilot-scale ultrasonic extraction equipment under optimal conditions. Compared with the traditional ethanol solution as the solvent method, the proposed method is safer and more environmentally friendly on the premise of ensuring the yield.

### CRediT authorship contribution statement

**Xinyu Yang:** Formal analysis, Investigation, Writing – original draft, Writing – review & editing, Funding acquisition. **Tingting Liu:** Formal analysis, Validation, Writing – original draft, Writing – review & editing, Methodology, Funding acquisition. **Shuwen Qi:** Investigation. **Huiyan Gu:** Conceptualization, Writing – review & editing. **Jialei Li:** Methodology. **Lei Yang:** Conceptualization, Writing – review & editing, Funding acquisition.

## Declaration of Competing Interest

The authors declare that they have no known competing financial interests or personal relationships that could have appeared to influence the work reported in this paper.
